# Tetrahydrobiopterin enhances regulatory T- and mast cell proliferation and alters cytokines expression in a murine heart transplant model

**DOI:** 10.1038/s41598-025-20127-1

**Published:** 2025-10-16

**Authors:** Susanne Ebner, Bernhard Texler, Florian Nardin, Maria R. Troppmair, Anh Vu Nguyen, Benno Cardini, Jakob Troppmair, Georg Schäfer, Gudrun C. Thalhammer-Thurner, Kerstin Nirtl, Katharina Lackner, Natalia Alenina, Dietmar Öfner, Stefan Schneeberger, Katrin Watschinger, Gerald Brandacher, Ernst R. Werner, Manuel Maglione

**Affiliations:** 1 Daniel Swarovski Research Laboratory, Department of Visceral, Transplant and Thoracic Surgery, Center of Operative Medicine, Medical University of Innsbruck, Innrain 66, 6020 Innsbruck, Austria; 2 Institute of Clinical and Functional Anatomy, Medical University of Innsbruck, Innsbruck, Austria; 3 Institute of Pathology, Neuropathology and Molecular Pathology, Medical University of Innsbruck, Innsbruck, Austria; 4 Institute of Molecular Biochemistry, Biocenter, Medical University of Innsbruck, Innsbruck, Austria; 5 Max Delbrück Center for Molecular Medicine in the Helmholtz Association (MDC), Molecular Biology of Peptide Hormones, Berlin, Germany

**Keywords:** Tetrahydrobiopterin, Immunosuppression, Regulatory t cells, Mast cells, Cytokines, Heart transplantation model, Allotransplantation, Translational research

## Abstract

Administration of tetrahydrobiopterin (BH4) has been shown to attenuate acute allograft rejection in a murine heart transplantation model in a manner similar to that of cyclosporine A. However, its mechanism of action on immune cells remains largely unknown. A fully MHC-mismatched (C3H/He to C57BL/6) mouse heart transplant model was used in this study. The recipients were treated with BH4 or cyclosporine A six days. The degree of acute rejection was assessed by histopathological analysis, splenocytes were analyzed by flow cytometry, and cytokine production was estimated based on the level of protein and RNA in sera and grafts and in vitro in T cell cultures. Proliferation of regulatory T cells and mast cells, suppressor capacity of Tregs, and MLR of T cells were conducted in vitro. Survival curves confirmed the significant improvement observed in the BH4-treated animals. BH4-treatment resulted in a substantial increase in Tregs and mast cells in the secondary lymphoid organs. In vitro assays showed increased proliferation of BH4-treated Tregs and mast cells. Cytokine production in vivo and in vitro in BH4-treated animals revealed an increase in the expression of IL-10, IL-5 and IL-4. BH4-dependent mast cell-derived tryptophan hydroxylase-1 could be excluded as a treatment target in recipient knockout mice. These data suggest that BH4 modulates the innate and adaptive immune systems, resulting in increased proliferation of regulatory T and mast cells accompanied by a modulation of anti-inflammatory cytokines.

**Supplementary Information:**

The online version contains supplementary material available at 10.1038/s41598-025-20127-1.

## Introduction

Advances in immunosuppressive medication have been crucial in transforming solid organ transplantation from an experimental approach to standard treatment of care^1^. However, successful maintenance immunosuppression strategies are affected by drug toxicities, limiting graft and recipient survival, and making the search for new immunosuppressive strategies/approaches indispensable^2–4^.

Tetrahydrobiopterin (BH4) exerts immunomodulatory effects in various solid organ transplantation models. In ischemia reperfusion injury (IRI) models, we demonstrated BH4-specific protection of the transplanted grafts, proposing BH4-dependent constitutive nitric oxide synthase (cNOS) as the drug target^5–8^. Similarly, the administration of exogenous BH4 to the donor prevented severe transplant vasculopathy, probably by attenuating IRI. cNOS was identified as the target. The stabilization of the dimeric isoform by BH4-administration was clearly associated with successful treatment^9^. However, the immunoregulatory properties of BH4 appear to go beyond NOS modulation and attenuation of IRI.

Using a mouse heart transplantation model, transgenic overexpression of the rate-limiting enzyme for BH4 synthesis GTP cyclohydrolase I in the transplanted graft, as well as exogenous administration of BH4 or its precursor sepiapterin, has been shown to attenuate acute allograft rejection to an extent similar to that of cyclosporine A (CsA)^10–12^. In contrast to IRI models, in this context, its direct effects on immune cells as well as the target molecule responsible for the immunosuppressive action of BH4 remain unexplored^12^. The BH4 dependent aromatic amino acid hydroxylase tryptophan hydroxylase-1 (TPH-1) has recently been shown to be involved in immune regulation by delaying graft rejection and promoting tolerance in a murine skin transplantation model. In this model, the major source of TPH-1 was recipient-derived mast cells (MCs) invading the transplanted skin^13^. MCs are involved in the adaptive immune response by processing and presenting antigens via MHC molecules. They ultimately link innate and adaptive immunity by secreting pro- and anti-inflammatory cytokines and mediators^14,15^.

Considering these findings, in the present study, we decided to further dissect the immunosuppressive properties of BH4, not only from a TPH-1/MC-related perspective but also, more generally, regarding its effects on immune cells in the setting of acute allograft rejection using a heart transplantation model in mice.

## Materials and methods

### Mice

Male C57BL/6 (H-2b) and C3H/He (H-2k) mice, aged 10–12 weeks were purchased from Charles River (Sulzfeld, Germany). TPH-1^**−/−**^ knock-out mice (C57Bl/6 background, ) were kindly provided by Prof. Dr. Michael Bader (Max-Delbrück-Centrum für Molekulare Medizin, Berlin, Germany) and were bred at the Central Animal Facility of the Medical University of Innsbruck. All animals were housed under standard conditions and received human care in compliance with the “Principles of Laboratory Animal Care” prepared by the National Academy of Sciences and published by the National Institutes of Health (NIH Publication No. 86 − 23, revised 1985). This study is reported in accordance with ARRIVE guidelines^16^. The Austrian Ministry of Education, Science, and Culture (BMWF-66.011/0061-V/3b/2019) approved all the experiments.

### Heart transplantation and experimental design

For the acute rejection model, C3H/He donor hearts were heterotopically transplanted into C57BL/6 mice (*n* = 5–7/group). Allogeneic cervical heart transplantations were performed under injection anesthesia with ketamine/xylazine (AniMedica, Senden-Bösensell, Germany) as previously described^17^ Briefly, the donor heart was flushed in situ with Custodiol (Dr. Franz Köhler Chemie) and procured en bloc, including the proximal aorta and the pulmonary artery. All other vessels were ligated, including the inferior vena cava, both superior venae cavae, and the pulmonary veins. The aorta of the graft was then connected to the right common carotid artery using a standardized non-suture cuff technique. Venous drainage was achieved via another cuff anastomosis between the donor’s pulmonary artery and the external jugular vein of the recipient. Allografts’ cold and warm ischemia times were maintained as short as possible by immediately transferring the graft into the recipient. Rejection was defined as complete cessation of ventricular contractions, and heart performance was scored as previously described^12^.

Survival of the allografts was assessed daily by palpation and binocular inspection. As previously described^12^, graft performance was scored according to a graft function score ranging from 0 to 4, where 0 represents complete cessation of heart beats and 4 represents unaltered function. Four different treatment groups were defined:^1^ non-treated, wild-type donor-recipient pairs;^2^ recipients treated with BH4 (50 mg/kg b.w. tid, i.m.);^3^ recipients treated with CsA (15 mg/kg b.w. OD, i.p.); and^4^ a syngeneic donor-recipient control group. According to our previously published protocol, recipient animals received either BH4 or CsA from day 0 to day 6 post-transplantation. All animals were sacrificed on day 6 post-transplantation for further analysis. On day six, the still beating hearts were harvested for further analysis before complete rejection^12^. Following the observation period, recipient animals were anesthetized with an intraperitoneal injection of xylazine (5 mg/kg b.w.) and ketamine (100 mg/kg b.w.) and died by exsanguination during blood and organ recovery for further analysis. Dosing regimens of the applied immunosuppressant’s as well as the administration period were chosen according to previously published work^12^.

**Fig. 1:**
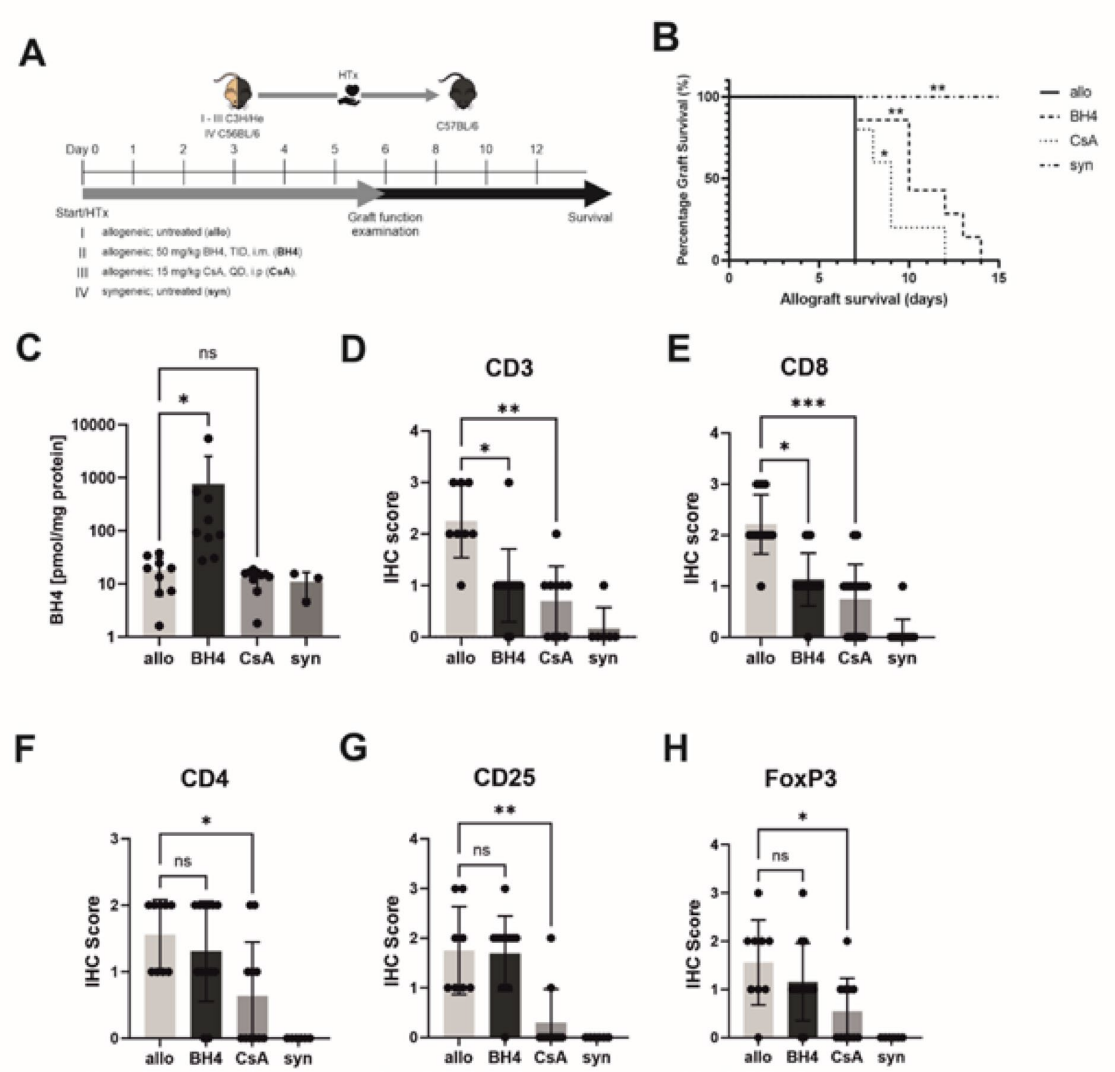
Treatment effects on cardiac allograft function, survival, and graft infiltrating T cells. (**A**) Experimental protocol/design of an allograft transplantation setting with BH4 and CsA treated mice. Survival of the graft was analyzed until day 15 and sample collection for IHC, FACS, RT-qPCR, Luminex and ELISA occurred on day 6. (**B**) Compared with untreated allogeneic control animals, BH4 treated recipients and CsA treated recipients showed significantly improved survival of heart grafts. *N* = 5–7 (**C**) Intragraft BH4 concentrations of BH4-treated animals were significantly increased compared to non-treated allogeneic controls and are presented as Mean ± SEM. *N* = 3–9 (**D**) CD3 infiltration of non-treated allogeneic control mice is significantly higher than in BH4-treated and CsA-treated allogeneic-transplanted mice. Histological staining revealed significantly lower frequencies of (**E**) CD8^+^ T cells in BH4- and CsA-treated animals compared to non-treated allogeneic control mice. Furthermore, we also identified similar numbers of graft infiltrating (**F**) CD4^**+**^ and (**G**) CD25^**+**^ lymphocytes in donor hearts of BH4-treated grafts compared to allogeneic non-treated transplanted hearts. CD4^+^ and CD25^+^ lymphocytes in CsA-treated transplanted hearts were significantly decreased. (**H**) Notably, there was no significant difference observed of FoxP3^+^ cells between BH4-treated animals and non-treated allogeneic controls. CsA-treated animals showed a significant decrease of FoxP3^+^ cells compared non-treated allogenic controls. The survival data were analysed by a Log-rank test and are presented as a Kaplan-Meier survival curve. Statistically significant differences between groups were tested applying the Kruskal-Wallis test with Dunn’s post-hoc test. In histological samples results are presented as Mean + SD. *N* = 6–13/group. Allogeneic and syngeneic controls were significantly different in all histological samples but not in BH4 concentrations (not depicted). No significant differences were observed in the expression of CD3, CD8 and CD4 between BH4- and CsA-treated mice. However, there was a significant difference in CD25 (*p* ≤ 0.01) and FoxP3 (*p* ≤ 0.05) expression (not depicted). Allo = untreated allogeneic controls, BH4 = BH4-treated allogeneic transplanted animals, CsA = CsA-treated allogeneic transplanted animals, syn = untreated syngeneic transplanted controls.

For survival analysis, the same experimental groups were used; however, the mice were observed until the ventricular contraction was stopped. (Fig. 1A) Euthanasia of this group of animals was performed by terminal isoflurane inhalation followed by cervical dislocation.

To test whether MC-derived TPH-1 in recipient animals was the target of BH4, four identical groups were analyzed using TPH-1^**−/−**^ mice as recipients.

People performing heart transplantation on mice and administering the different treatments were of involved in data analysis of the recovered specimens.

### Flow cytometry

For flow cytometry analysis, on day six following transplantation, the mouse spleens were minced, and splenocytes isolated using a cell strainer with 40 μm diameter pores and washed twice with PBS. Red blood cells were lysed with the RBC Lysis Buffer (Thermo Fisher Scientific, Waltham, MA, USA), and the samples were analyzed after staining with 7AAD (BD Bioscience, Heidelberg, Germany). Typically, 1 × 10^6^ cells were pre-incubated with FcR-blocking reagent (BD Bioscience, Heidelberg, Germany) for 15 min and afterward stained with combinations of antibodies listed in Supplemental Table 1. For the FoxP3 intracellular staining, the cells were stained with Fixable Viability Dye (Thermo Fisher Scientific, Waltham, MA, USA), fixed and permeabilized with the FoxP3/Transcription Factor Staining Buffer Set (Thermo Fisher Scientific, Waltham, MA, USA), and incubated with Normal Rat Serum (Thermo Scientific, Waltham, MA, USA) prior to the incubation with the FoxP3 antibody. Cells were analyzed on a BD LSRFortessa flow cytometer (BD Bioscience, Heidelberg, Germany). For analysis, only living cells were counted. Moreover, doublet exclusion was achieved by plotting forward and sideward scatter areas, heights, and widths (FSC- and SSC-A/-H/-W). Data were analyzed with FlowJo v 6.2 and FlowJo v 10.7 (Tree Star, Ashland, OR). The gating strategy is presented in Supplemental Figs. 5 and 6.

### Proliferation assays

For regulatory T cells (Tregs), splenocytes of male C57BL/6 mice aged 10 to 12 weeks were obtained from spleens as described in the chapter flow cytometry. Naive CD4 T cells were enriched from pooled splenocytes via magnetic-activated cell sorting (MACS) (Miltenyi Biotec, Bergisch Gladbach, Germany) according to the manufacturer’s instructions. Naïve CD4 T cells were incubated in RPMI containing 10% FCS with 135 U/ml human recombinant IL-2, 20 ng/ml TGF-β (both Thermo Fisher, PeproTech, Waltham, MA, USA) and 1 nM Retinoic acid (St. Louis, MO, USA) in a 24-well plate precoated with 10 µg/ml mouse α-CD3 antibody (BioLegend, San Diego, CA) to get induced Tregs (iTregs) for 4 days. On day 4 Tregs were stained with CFSE according to the protocol of Quah et al.^18^. 5 × 10^5^ labeled iTregs were incubated stimulated (10 µg/ml CD3 antibody, 2 µg/ml α-CD28 antibody. BioLegend, San Diego, CA) and unstimulated with and without sepiapterin (1 µM) in 96-well U-bottom plates for 3 days. The sepiapterin dose was determined using a concentration series (0.1, 1 and 10 µM), as specified in the data sheet. Based also on our previous experience, 1 µM was ultimately used^19^. Cells were analyzed on a BD LSRFortessa flow cytometer (BD Bioscience, Heidelberg, Germany). Data were analyzed with FlowJo v 10.7.0 (Tree Star, Ashland, OR). The gating strategy is presented in Supplemental Fig. 7.

The FlowJo proliferation platform was used to analyze the Proliferation Index. This platform is a specialized tool within the FlowJo software designed for analyzing cell division data obtained from flow cytometry experiments using cell-tracking dyes like CFSE or CTV. It quantifies cell proliferation by modeling the distribution of dye dilution as cells divide. This platform provides graphical displays, statistical summaries, and automated gating to differentiate between cell generations. FlowJo uses mathematical models to fit the observed dye dilution patterns, allowing for the determination of cell division events and their frequency. The platform calculates various proliferation statistics, including the Proliferation Index (the average number of divisions for the dividing population), the Division Index (the average number of divisions for all cells in the original culture), the Expansion Index (the fold expansion of the original population, taking into account all cells, including those that divided and those that did not) and the Replication Index (the average number of cells generated per original dividing cell, considering only cells that divided).

For MCs, we performed peritoneal lavage of wild-type male C57BL/6 mice with PBS and collected the MC precursors. Mature MCs were generated by seeding 2 × 10^6^ cells in a T25 flask, adding 30 µg/ml SCF and 10 µg/ml IL-3 according to the protocol described by Vukman.

et al.^20^. In the Treg and MC proliferation assays, the BH4 precursor sepiapterin (Schircks Laboratories, Jona, Switzerland) was added on days 3 and 6, and proliferation was analyzed by counting the number of cells using trypan blue.

### Suppressor assay

Tregs and naïve CD4^**+**^ T cells (responder T cells) were obtained as in the proliferation assay. Antigen-presenting cells (APCs) were obtained via MACS as the negative fraction of CD90.2 Microbeads (Miltenyi Biotec, Bergisch Gladbach, Germany) from C57/BL/6 mice. CD4^**+**^ T cells were stained with CFSE as responder T cells according to the protocol of Quah et al. Tregs were stained with cell tracer violet (Thermo Fisher, Invitogen, Waltham, MA, USA) according to the manufacturer’s instructions. We plated with and without Sepiapterin (1 µM) 1 × 10^5^ responder T cells and 1 × 10^5^ irradiated APC (15 Gy) in a U-bottom 96-well plate with decreasing concentrations of Tregs (1:1, 1:2, 1:4, 1:8, 1:16) in 250 µl of RPMI with 10% FCS for 3 days. 1 µg/ml anti-CD3 antibody (BioLegend, San Diego, CA) was added per well. Cells were analyzed on a BD LSRFortessa flow cytometer (BD Bioscience, Heidelberg, Germany). Data were analyzed with FlowJo v 10.7.0 (Tree Star, Ashland, OR). The gating strategy is presented in Supplemental Fig. 7.

### Mixed lymphocyte reaction

Naïve CD4^**+**^ T cells from C57BL/6 mice and APCs from C3H/He (H-2k) mice were obtained as in the proliferation assay. 1 × 10^**5**^ APCs were irradiated with 15 Gy and plated with 1 × 10^**5**^ CTV-stained responder T cells in a 96-well U-bottom-plate for 3 days. 1 µg/ml anti-CD3 antibody (BioLegend, San Diego, CA) was added per well. Cells were plated without and with Sepiapterin (1 µM). Cells were analyzed on a BD LSRFortessa flow cytometer (BD Bioscience, Heidelberg, Germany). Data were analyzed with FlowJo v 10.7.0 (Tree Star, Ashland, OR). The gating strategy is presented in Supplemental Fig. 7.

### Cytokine detection

For Luminex assays and ELISA, on day six after transplantation, sera from indicated recipients were collected and stored at -20 °C. IL-2, IL-4, IL-6, IFN-γ, and TNF-α were measured with ProcartaPlex™ multiplex, high sensitivity Immunoassay, and TGF-β, IL-5, IL-13, IL-17a, IL-21, IL-22, IL-33, and TIM-3 were measured with ProcartaPlex™ simplex kits according to the manufacturer´s instructions (both Thermo Fisher Scientific, Invitrogen, Waltham, MA, USA). Cytokine levels of IL-9 were analyzed by high sensitivity sandwich ELISA performed according to the manufacturer’s instructions. (Abcam, Cambridge, England). For in vitro assays, IL-10 was measured with DuoSet^®^ Mouse IL-10 (R&D Systems, Minneapolis, MN, USA) and IL-2, IFN-γ, IL-4, IL-5, IL-13 were measured with ProcartaPlex™ Mo Th1/Th2 & Chemokine Panel 1 20plex according to the manufacturer´s instructions (both Thermo Fisher Scientific, Invitrogen, Waltham, MA, USA).

### Quantitative reverse transcription polymerase chain reaction

Total RNA from the snap-frozen heart tissue was extracted using the RNAII Kit (Macherey-Nagel, Düren, Germany), according to the manufacturer’s instructions. The quantification and purity of the extracted RNAs were assessed using a Thermo Scientific Nano-Drop 2000 spectrophotometer (Brookfield, Wisconsin, USA). Real-time reverse transcription polymerase chain reaction (RT–PCR) for gene expression analysis was performed with the ABI PRISM 7500 Sequence Detection System (Life Technologies, Carlsbad, CA, USA). Primers were purchased directly as Taqman^®^ gene expression assays (Thermo Fisher Scientific, Waltham, MA, USA) (Supplemental Table S2). The PCR reaction was performed in a final volume of 25 µl containing 1 µl of cDNA, 12.5 µl of Master Mix (Thermo Fisher Scientific, Waltham, MA, USA), 1 µl of fluorogenic hybridization probe, 6 µl of primer mix, and 5.5 µl of distilled water. The amplification consisted of a two-step PCR (40 cycles; 15 s denaturation step at 95 °C and 1 min annealing/extension step at 60 °C). The mean CT values were calculated from double determinations (excluding deviations > 0.5 cycles), and samples were considered negative if the CT values were > 40 cycles. Specific gene expression was normalized to the housekeeping gene hypoxanthine-guanine phosphoribosyltransferase (HPRT), given by the formula 2^–ΔCT^.

### Histology and immunohistochemistry

Tissues were fixed in 4% neutral buffered formalin for 12 h at room temperature, embedded in paraffin; 2 μm sections were stained with hematoxylin and eosin (H&E) and scored according to the standards of the International Society for Heart and Lung Transplantation (ISHLT) rejection score (no – mild – moderate – severe rejection)^21^. For immunohistochemistry, 2 μm paraffin tissue sections were stained manually using the listed rabbit anti-mouse monoclonal primary antibodies (CD3 Abcam, ab16669, 1:50; CD4 Cell Signaling, #25229, 1:100; CD8 Cell Signaling, #98941, 1:50; CD25/IL-2Rα Cell Signaling, #36128, 1:100; FoxP3 Cell Signaling, #12653, 1:400) for 1 h after heat mediated antigen retrieval (citrate buffer in an autoclave for 10 min). The visualization was carried out with a Dako EnVision^+^ System- HRP Labelled Polymer kit (K4003) for 30 min. All steps were carried out at room temperature. Stained slides were digitally scanned by a Panoramic 250 Flash III scanning system (3DHISTECH, Hungary) and evaluated on digital slides using the CaseViewer software (3DHISTECH, version 2.2, www.3dhistech.com, Hungary). Scoring was performed in hotspots in high magnification (63x) according to the number of positive cells in the tissue area displayed by the monitor, with a score from 0 to 3.

### Biopterin measurements by high performance liquid chromatography (HPLC)

Intragrafted BH4 concentrations were measured using the method described by Fukushima and Nixon^22^. BH4 concentrations were calculated as the difference in biopterin concentrations under acidic and basic oxidation conditions. Dihydrobiopterin plus biopterin (BH2 + B) concentrations were calculated as the difference between the total intragraft biopterin and intragraft BH4 concentration.

### Statistical analysis

GraphPad Prism 9.4.1 software (GraphPad, La Jolla, CA, USA) was used for statistical analysis of the data. Before using parametric statistical tests, the required assumption of normality was tested using the Shapiro-Wilk test. Depending on the normal distribution, ANOVA (with Dunnett´s post-hoc test) or Kruskal-Wallis test (with Dunn´s post-hoc test) were chosen for multiple comparisons. For two-group comparisons, t-test or Wilcoxon test (depending on data distribution; for paired datasets) or t-test or Mann-Whitney test (depending on data distribution; for unpaired datasets) was used. The levels of significance: *p* ≤ 0.05 (*), *p* ≤ 0.01 (**) and *p* ≤ 0.001 (***) and *p* ≤ 0.0001 (****).

## Results

### BH4-treatment prolongs graft survival

First, we examined the effects of BH4 and CsA treatments on cardiac allograft survival. The untreated allografts stopped beating on day seven post-transplantation, whereas all the syngeneic grafts survived for more than 15 days. The Kaplan-Meier survival curve indicated significantly prolonged survival of allografts in BH4-treated mice (*p* = 0.01) and CsA-treated mice (*p* = 0.05) compared to untreated allogeneic grafts (Fig. 1B). BH4 administration resulted in a significant increase in BH4 levels in the sera of BH4-treated mice compared to all other recipient animals (Fig. 1C).

### BH4-treatment modulates T cell populations infiltrating the graft

We evaluated the infiltration of CD3^**+**^ T cells into the grafts by histology. In line with less severe tissue destruction, we revealed significantly reduced CD3^**+**^ T lymphocyte infiltration after BH4 and CsA treatment compared to the allogeneic control (Fig. 1D). Next, we analyzed the subtypes of CD3^+^ T cells, such as CD8^+^ and CD4^+^ cells and determined CD25, and FoxP3 expression in the grafts (Fig. 1E, F, G, H and Supplemental Fig. 1). The IHC score of CD8 was significantly decreased in both, BH4- and CsA-treated animals compared to that in the allogeneic control. The IHC scores of CD4, CD25, the alpha chain of interleukin 2 receptor, and FoxP3, the transcription marker of Tregs, showed no significant difference between allogeneic-transplanted controls and BH4-treated animals but were significantly decreased in CsA-treated animals.

The most prominent cellular infiltrates and myocyte damage were seen in allogeneic transplanted grafts. These infiltrates were significantly attenuated in CsA-treated animals, whereas BH4 treatment resulted in lower, but not statistically significant, ISHLT scores compared to allogeneic controls (Supplemental Fig. 2).

### BH4-treatment is associated with increased frequencies of regulatory T cells and mast cells in lymphoid organs

**Fig. 2:**
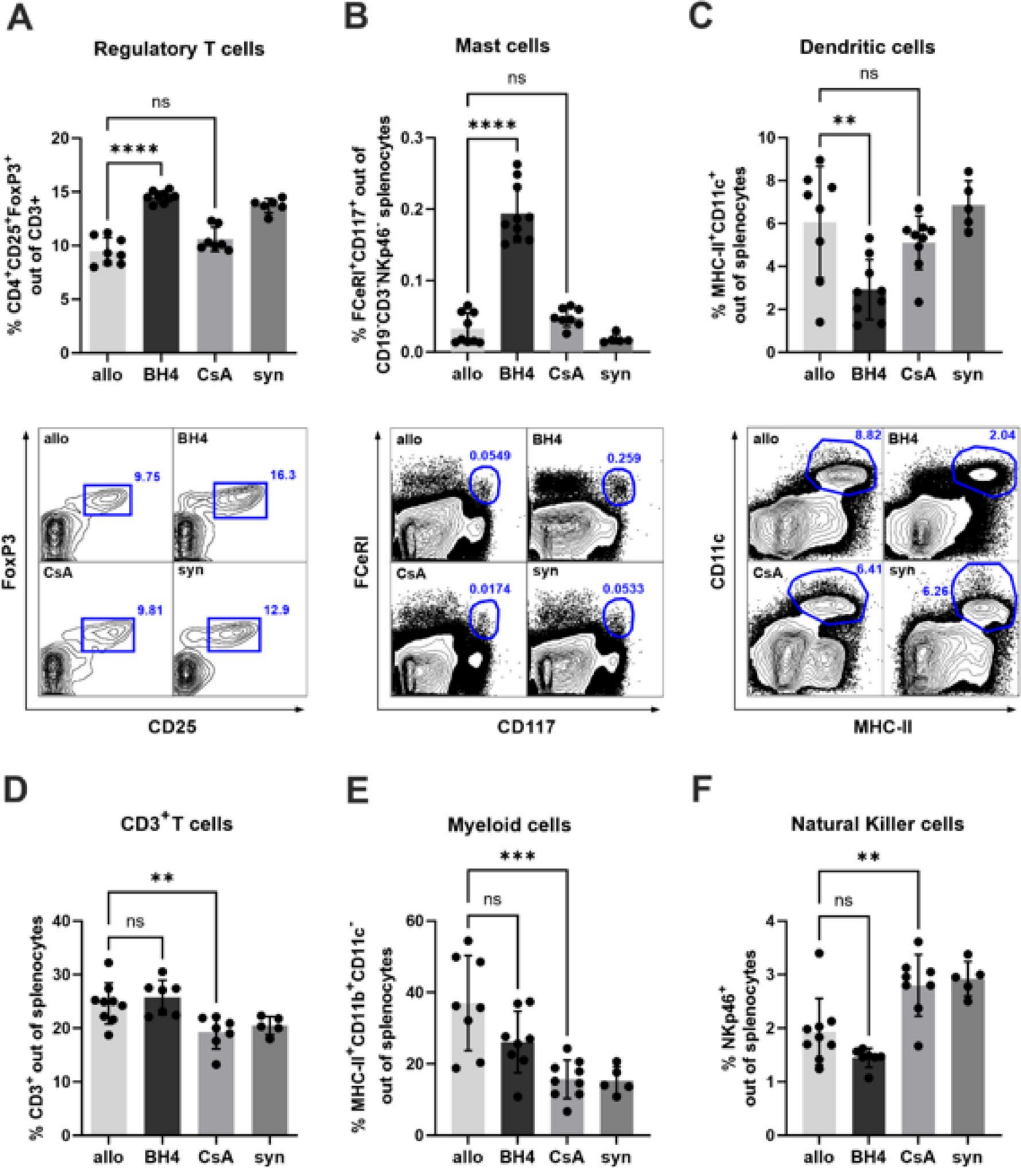
Treatment effects on immune cell mobilization. Compared with untreated allogeneic control mice significantly higher frequencies of (**A**) regulatory T cells and (**B**) mast cells were detected in spleens derived from recipients treated with BH4 at day 6 post-transplantation. (**C**) Frequency of DCs was decreased in BH4-treated animals compared to untreated allogeneic control mice. (**D**) CD3^**+**^ T cells, (**E**) CD11b^**+**^CD11c^**−**^ myeloid cells and (**F**) NK cells in the spleen showed no statistical difference between the BH4-treated mice and non-treated allogeneic control mice. Non-treated allogeneic control mice showed a statistical difference with CsA-treated mice. Significant differences were observed between CsA- and BH4-treated mice in all six analyzed populations (**A**–**F**). The p values are as follows: Tregs and MCs, *p* ≤ 0.0001; NK cells, *p* ≤ 0.001; CD3 T cells, *p* ≤ 0.01; myeloid cells and DCs, *p* ≤ 0.05 (not depicted). Statistically significant differences between groups were tested applying the one-way ANOVA test with a Dunnett´s multiple comparison test. *N* = 6–10/group. Allogeneic and syngeneic controls were significantly different in A, D and E but not in B, C and D (not depicted). Treg = regulatory T cells, MCs = mast cells, DCs = dendritic cells, NK = Natural Killer. Exact gating strategy is shown is supplemental Figs. 5 and 6.

Compared to untreated controls and CsA-treated animals, CD4^+^ CD25^+^ FoxP3^+^ Tregs and MCs were significantly increased in the spleen (Fig. 2A, B) and draining lymph nodes (Supplemental Fig. 3) of BH4-treated recipients.

In contrast, BH4 treatment significantly reduced the frequency of myeloid dendritic cells (DCs) in the spleen (Fig. 2C) while we did not observe changes in CD3^+^ T cells, NKp46^+^ NK cells, and CD11b^+^CD11c^**−**^ myeloid cells in BH4-treated mice compared to untreated controls (Fig. 2D, E, F). Compared to untreated controls, CsA-treatment resulted in a significant decrease in the frequency of CD3^+^ T cells and NKp46^+^ NK cells, as well as in a significant increase in the frequency of myeloid CD11b^+^CD11c^**−**^ cells (Fig. 2D, E, F).

### BH4-treatment induces proliferation of regulatory T cells but does not increase suppressor capacity

For the in vitro experiments, we used the BH4-precursor sepiapterin (Schircks Laboratories, Jona, Switzerland) instead of BH4. First, because it is known to be superior to BH4 in increasing cellular BH4 levels^23^, and second, because of the higher partial pressure of O^2^ in standard cell cultures, BH4 is auto-oxidized and H_2_O_2_ is formed, resulting in BH4 independent effects that can be inhibited by catalase^24^. In contrast, sepiapterin is taken up by cells and converted to BH4, which requires sepiapterin reductase and dihydrofolate reductase for its conversion^25^.

**Fig. 3:**
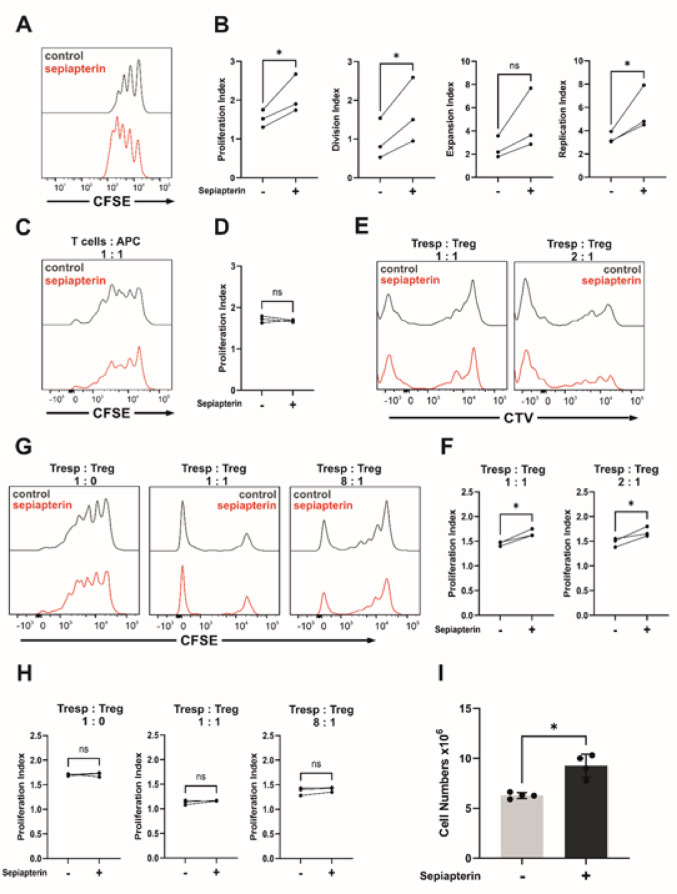
In vitro proliferation assay, suppressor assay and mixed lymphocyte reaction (**A**,**B**) Proliferation analysis of sepiapterin-treated (red) or control (black) murine induced regulatory T cells showing CFSE staining patterns of FoxP3^+^ T cells. Herein, we observed an increased proliferation under sepiapterin treatment displayed by a representative CFSE histogram (**A**) as well as using the proliferation index, division index, expansions index and replication index (**B**). Sepiapterin concentration was 1µM. (**C**,**D**) Proliferation analysis of sepiapterin-treated (red) and control (black) CD4^+^ T cells of C57BL/6 mice in a mixed lymphocyte reaction with C3H/He antigen presenting cells (APCs) as responder cells. Sepiapterin treatment had no impact on the activation of CD4^+^ T cells shown by the CFSE histogram (**C**) and the calculated proliferation index (**D**). (**E**,**F**) Proliferation analysis of sepiapterin-treated (red) or control (black) regulatory T cells during a T cell suppressor assay using cell tracer violet (CTV) stained regulatory T cells (Tregs) and CFSE stained responder T cells (Tresp). Left peak in the graphs are the CFSE stained responder T cells and the right peak depicts the regulatory T cells, which displayed an increased proliferation under sepiapterin treatment shown in the histogram (**E**) as well as using the proliferation index (**F**). While the Tregs efficiently suppressed the Tresp, sepiapterin treatment did not result in any changes as seen in the histograms (**G**) or in the calculated proliferation indices (**H**). T cell suppression analysis of sepiapterin-treated (red) or control (black) regulatory T cells showing CFSE staining patterns of responder T cells (Tresp). While the Tregs efficiently suppressed the Tresp, sepiapterin treatment did not result in any changes (**G**, **H**). Left peak in 1:1 and 8:1 graphs are the CTV stained Tregs and the right peak depicts Tresp. (**I**) Proliferation of peritoneal-derived mast cell precursors after 10 days of culture with and without sepiapterin. Initially 2 × 10^6^ cells were seeded in a 25 cm^2^ flask. On day 10 total amount of cells was counted with trypan blue. Sepiapterin concentration = 1µM. Differences between control and sepiapterin-treated regulatory T cells (*n* = 3/group) and sepiapterin-treated mast cells (*n* = 4/group) were calculated by paired t tests.

In the in vitro proliferation assay, we showed that the proliferation of sepiapterin-treated Tregs was increased compared to that of untreated controls (Fig. 3A, B). However, during MLR, the proliferation of sepiapterin-treated CD4^+^ T cells was unchanged compared to that of the control cells (Fig. 3C, D). Sepiapterin treatment enhanced the proliferation of Tregs but not that of CD4^+^ T cells in an MLR.

Additionally, we investigated whether sepiapterin induces the suppressive capacity of Tregs. Tregs stained with CTV showed increased proliferation under sepiapterin treatment compared to the controls (Fig. 3E, F, Supplemental Fig. 8). However, in no ratio of Tregs to responder T cells we observed a modulation of suppression of the activity of the responder T cells due to sepiapterin (Fig. 3G, H).

### BH4 induces proliferation of mast cells

We carried out an in vitro proliferation experiment where we counted with trypan blue after ten days of culture the amount of MCs generated from MC precursors with and without sepiapterin. As shown in Fig. 3I, MCs generated with sepiapterin significantly increased their cell numbers compared to untreated controls.

### BH4-treatment modulates cytokine expressions

To assess whether serum cytokine levels changed after BH4-treatment compared with untreated allogeneic controls, we collected sera from recipient mice on postoperative day 6. To examine cytokine expression in the transplanted grafts, we performed RT-PCR.

**Fig. 4:**
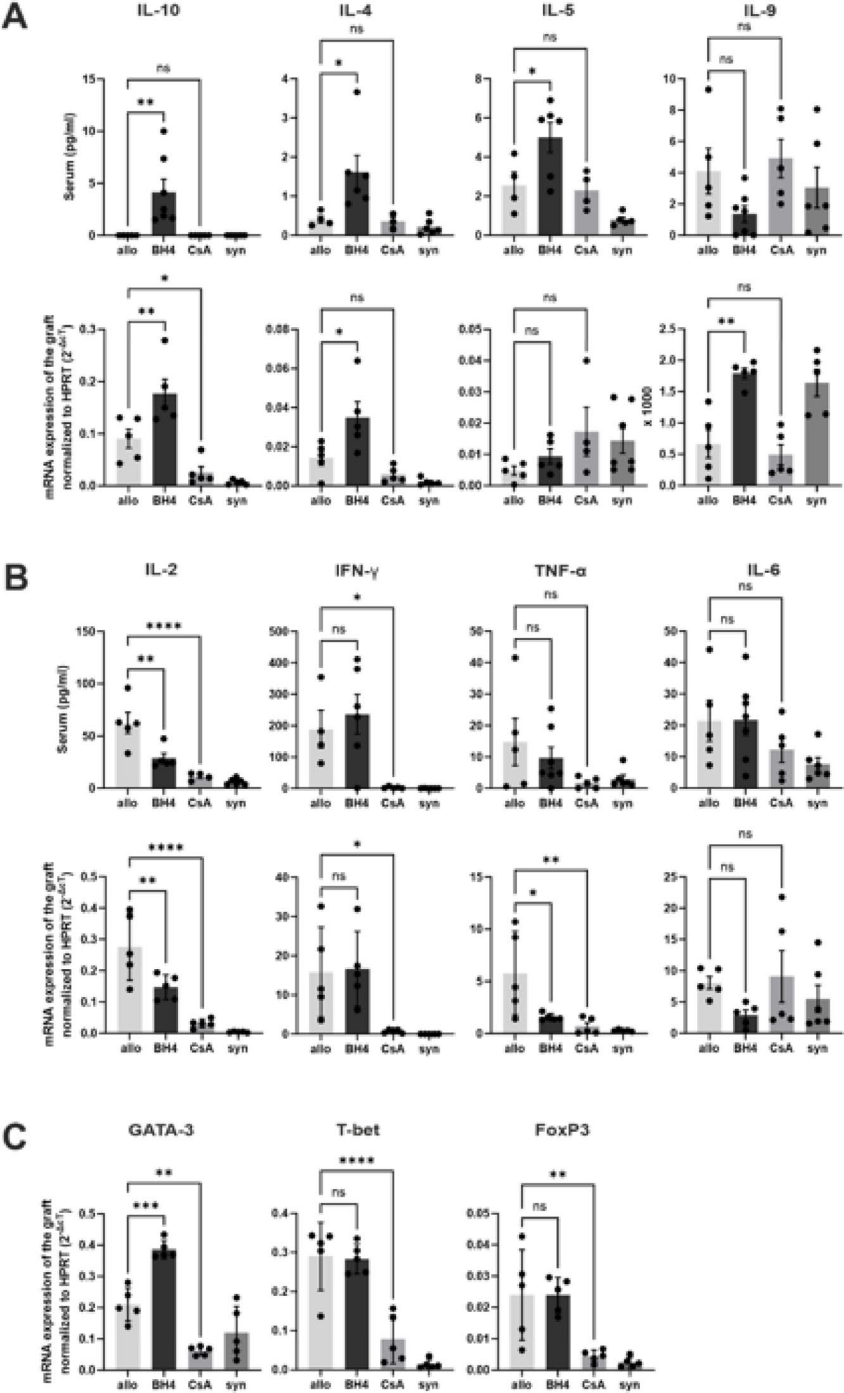
Treatment effects on transplanted hearts and serum cytokine levels. (**A**) In sera cytokine levels of IL-10, IL-4 and IL-5 were significantly increased following BH4 treatment. IL-9 showed no differences between the groups. Assessment of intragraft mRNA profile revealed a significant increase of IL-10, IL-4 and IL-9 but not of IL-5 in BH4 treated animals compared to untreated allogeneic controls. CsA-treated animals displayed no significant changes in their cytokine expression neither in sera nor in intragraft mRNA profile compared to untreated allogeneic controls. Only IL-10 is significantly downregulated in the transplant in CsA-treated animals compared to untreated allogeneic controls. (**B**) The pro-inflammatory cytokine IL-2 was significantly reduced in the sera and in the intragraft mRNA profile of BH4-treated and CsA-treated transplants. IFN-γ appeared unchanged in both serum and intragraft mRNA profiles in BH4-treated mice compared to untreated allogeneic-transplanted mice in contrast to CsA-treated animals. TNF-α of intragraft mRNA profile was significantly decreased in the BH4-treated group and CsA-treated group compared to untreated allogeneic mice, however, this was not reflected in sera of BH4-treated and Csa-treated mice. IL-6 expression in BH4-treated and CsA-treated animals was unchanged compared to untreated allogeneic controls in intragrafts and in sera. (**C**) Transcription factor GATA-3 was significantly upregulated in BH4 treated animals compared to untreated allogeneic control mice and significantly donwnregulated in CsA-treated mice compared with untreated allogeneic controls. Furthermore, T-bet showed no difference in BH4 treated animals compared to untreated allogeneic controls but significant difference to CsA-treated animals. FoxP3 mRNA expression in BH4 treated mice is in the same range as in untreated allogeneic transplanted animals, but significantly downregulated in CsA-treated animals. Significant differences were observed between CsA- and BH4-treated mice. The p values are as follows: Gata-3, *p* ≤ 0.000.1; T-bet, *p* ≤ 0.00.1; FoxP3, *p* ≤ 0.05 (not depicted). Statistically significant differences between groups were tested applying the one-way ANOVA test with a Dunnett´s multiple comparison test. Results are presented as Mean + SD. *N* = 4–6/group. Allogeneic and syngeneic controls were significantly different in sera of IL-2, IFN- γ and in intragraft mRNA profiles of IL-10, IL-9, IL-2, IFN- γ, TNF-α, GATA-3, T-bet and FoxP3 (not depicted).

BH4-treated mice showed significantly increased production of the anti-inflammatory cytokine IL-10 in their sera compared to the allogeneic control. This is reflected in the transplanted grafts. Moreover, significantly increased production of IL-4 and IL-5, two classical TH2 cytokines, was detected in the sera of BH4-treated animals compared to non-treated controls. In addition, IL-4 expression was significantly upregulated in grafts. Expression of IL-9, a cytokine also produced by Tregs and TH2 cells, was significantly upregulated in the grafts of BH4-treated mice compared to that in control animals (Fig. 4A).

Pro-inflammatory cytokines exhibit a more complex pattern. While IFN-γ in the serum and transplanted organs of BH4-treated mice did not differ from controls, TNF-α was significantly downregulated in the transplanted organs of BH4-treated animals. Moreover, in transplanted grafts of BH4-treated mice IL-6 expression tended to be less pronounced. IL-2 secretion in the sera of BH4-treated mice was significantly downregulated compared with that in allogeneic control mice. This was reflected in the grafts of BH4-treated animals but not to the same extent. CsA-treated mice displayed almost no IL-2 in their serum or graft (Fig. 4B).

In Fig. 4C, the mRNA expression levels of the transcription factors GATA-3 (TH2), T-bet (TH1), and FoxP3 (Tregs) in grafts are summarized. GATA-3 expression was upregulated in BH4 treated animals compared to that in CsA-treated and non-treated mice. T-bet and FoxP3 expression in BH4-treated animals was in the range of allogeneic controls, but was consistently higher than that in CsA-treated animals.

**Fig. 5:**
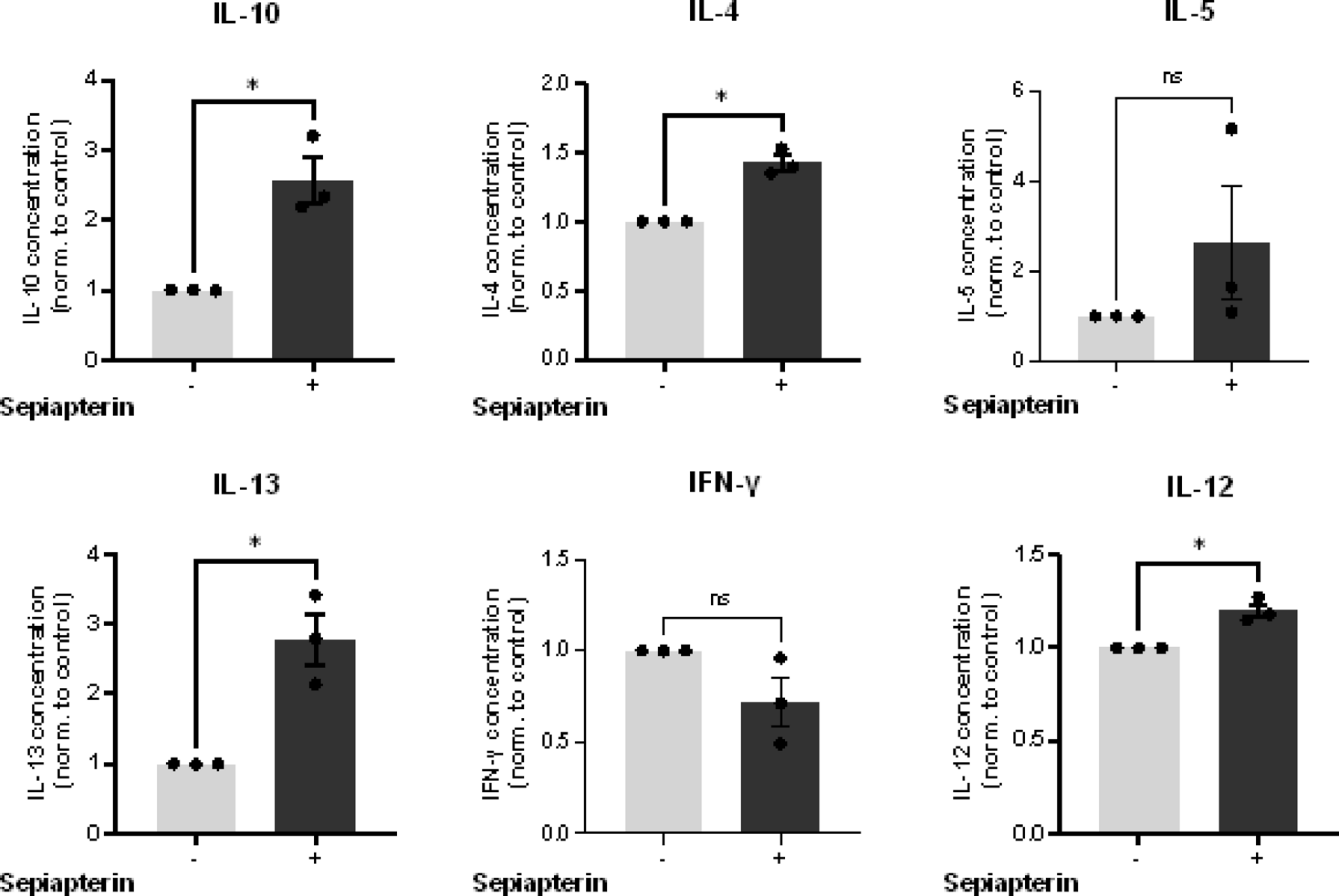
Cytokine expression in an in vitro assay with αCD3/αCD28 stimulated pan T cells with and without sepiapterin treatment. Cell culture supernatants were analyzed on day 3 of stimulation. 1µM sepiapterin was used. Cytokine levels of IL-4, IL-12, IL-13 and IL-10 were increased following sepiapterin treatment. IFN-γ and IL-5 showed no difference between the groups. Mean with SEM is shown. Statistically significant differences between groups were tested applying a paired t-test. *N* = 3 independent experiments.

The cytokine expression in pan T cells in an in vitro assay with and without sepiapterin is shown in Fig. 5. We did not observe a change in the TH1 cytokine INF-γ in sepiapterin-treated cells compared to that in untreated controls, but there was a slight increase in IL-12 p70 production. Of note, TH2 cytokines, IL-4, IL-13, and the immunosuppressive cytokine IL-10, were significantly upregulated in sepiapterin-treated cells. IL-5 showed a trend toward sepiapterin-treated cells producing more IL-5. However, this trend was not significant.

### BH4-treatment does not prolong graft survival in TPH1^−^/ ^−^ mice

**Fig. 6:**
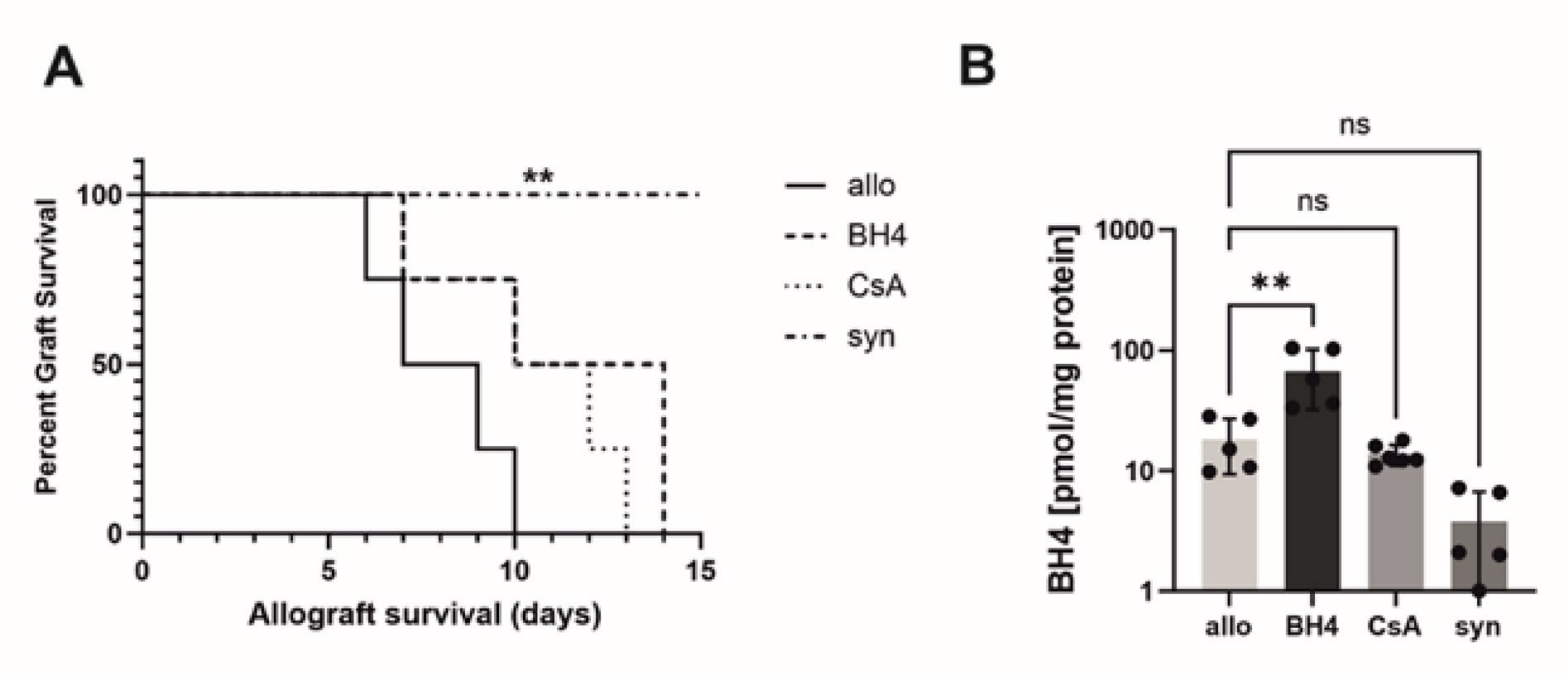
Treatment effects on cardiac allograft survival of THP-/- mice and intragraft BH4 concentrations. (**A**) Compared with untreated allogeneic control animals, BH4 treated recipients and CsA treated recipients showed no significantly improved survival of heart grafts. *N* = 5 (**B**) Intragraft BH4 concentrations of BH4-treated TPH-/- mice were significantly increased compared to non-treated allogeneic TPH-/- mice and are presented as Mean ± SEM. *N* = 5. The survival data were analysed by a Log-rank test and are presented as a Kaplan-Meier survival curve. Statistically significant differences between groups were tested applying the Kruskal-Wallis test with Dunn’s post-hoc test.

In view of the observed increased frequency of MCs, we transplanted wild-type grafts into TPH1^−/−^ recipient mice to test whether MC-derived TPH1 could emerge as a treatment target for BH4. As illustrated in Fig. 6A, wild-type hearts transplanted into TPH1^−/−^ recipient mice stopped beating on day 8 (median). Similarly, in the wild-type setting, TPH1^−/−^ recipient mice treated with either BH4 or CsA experienced increased graft survival compared with non-treated recipients; however, this difference was not statistically significant. All syngeneic grafts displayed indefinite survival. BH4 levels in TPH1^−/−^ mice were controlled by HPLC and were clearly increased (Fig. 6B).

## Discussion

In this study, we confirmed the immunosuppressive effect of BH4 in a solid organ transplantation model and elucidated distinct mechanistic differences with regard to immunomodulation when compared to the calcineurin inhibitor CsA. BH4-treatment results in (I) increased expression of FoxP3 in the allograft, (II) increased frequencies of Tregs in the spleen and lymph nodes, (III) increased proliferation of Tregs in vitro, (IV) increased levels of IL-10, IL-4 and IL-5 and (V), increased frequency of MCs in the spleen and in vitro. Finally, (VI) our data do not support MC-derived TPH-1 as a treatment target for BH4.

A recent study described BH4 as a critical regulator of T cell biology and suggested that BH4 boosted the proliferation of T cells, IL-2 secretion, and autoimmune responses^26^. Here we see that allografts of BH4-treated animals result in a longer survival compared to allogeneic controls, which refers to a previously described potent immunosuppressive effect of BH4^10,11,24^ rather than to a general „immune boost”. This is reflected by a lower amount of IL-2 in the transplanted grafts and sera in BH4-treated recipients than in allogeneic controls and by a clearly lower amount and frequency of infiltrating T cells found in the allografts. The observed mRNA expression of FoxP3 in the allograft, as well as the increased numbers of Tregs in the spleen and lymph nodes of BH4-treated recipients, could possibly support this interpretation. IHC showed that infiltration of FoxP3^+^ was unchanged in BH4-treated animals compared to untreated allogeneic controls; however, CD3^+^ T cells were significantly lower. This might reflect an indirect immunosuppressive effect of BH4 which is different from the calcineurin inhibitor CsA, culminating in a change in the proportion of different T-cell subsets infiltrating the allograft. If BH4 treatment would preferentially expand Tregs in a transplant model, as our data suggest, this could create a suppressive microenvironment that limits the accumulation of CD8 + cells in the graft. In this regard, enhancing Tregs numbers has been observed to result in reduced infiltration of effector T cells^27–29^.

Similarly, we demonstrated Treg-specific modulation of BH4. In vitro, the proliferation of sepiapterin-treated Tregs was increased compared to untreated controls, and during an MLR with CD4^+^ T cells proliferation of sepiapterin-treated CD4^+^ T cells was unchanged compared to control cells. It can therefore be concluded, that CD4^+^ T cells, in contrast to Tregs, are not stimulated to proliferate by sepiapterin, at least in the specific situation of an MLR. In this study we cannot provide any evidence that sepiapterin acts directly on Tregs or on naïve CD4 + T cells specifically. It has been, however, observed that any immune modulation by sepiapterin likely requires or follows T cell activation. Therefore, CD3- and CD28-activated naïve T cells or their metabolites could mediate the effects on iTregs^26,30^.

Furthermore, we were able to demonstrate increased Treg proliferation under BH4-treatment compared to controls, without influencing the suppressive capacity of BH4-treated Tregs. A possible explanation for BH4 differently stimulating Tregs and effector T cells could be their different metabolism, fatty acid oxidation, and aerobic glycolysis^31^. A recent study reported that reduced BH4 levels in cardiomyocytes resulted in the downregulation of genes involved in fatty acid metabolism and the upregulation of genes associated with glycolysis. The NO-independent effect of BH4 in macrophages was further characterized by a decrease in the protein levels of key enzymes involved in fatty acid oxidation, such as acyl-CoA synthetase and acyl-CoA dehydrogenase^32^.

The importance of this observation relies on the fact that active suppression by Tregs, which drives mechanisms such as the modulation of DC function, inhibitory cytokine release, cytolysis, and metabolic disruption, is crucial for the induction and maintenance of self-tolerance and immune hypo-responsiveness towards the allograft^33,34^. Tregs can prevent allograft rejection and the development of immunosuppressive drugs selectively promoting Tregs generation and function is an approach that can inhibit the activity of effector T cells^35^.

Recently, several groups revealed that innate immune cells, including MCs, play a pivotal role in allograft rejection^36–39^. and migrate from the graft to the draining lymph nodes during the early phase of organ transplantation^40^. In this regard, it is interesting to observe that the frequency of MCs is also significantly increased in the spleens and lymph nodes of BH4-treated animals compared to untreated allogeneic controls. Correspondingly, our in vitro data showed an increased proliferation of MCs following sepiapterin treatment. Unfortunately, MC staining within transplanted organs did not result in a reliable score. Based on these data and the observation that the MC-derived BH4-dependent aromatic amino acid hydroxylase, TPH-1, is involved in tolerance induction^13^, we hypothesized that TPH-1 could be the target of BH4 in our model.

However, although TPH1-/- recipient mice treated with BH4 showed increased graft survival compared to untreated recipients, the difference was not significant. In this regard, a TPH-1 independent effect of BH4 on MCs cannot be excluded and will be the focus of future research efforts.

In our study, the frequency of DCs in the spleens of BH4-treated animals was significantly reduced compared with that in untreated allogeneic controls. This was also observed in the BH4-treated animals that did not undergo transplantation (Supplemental Fig. 4). Furthermore, CsA-treated recipients displayed a slightly reduced frequency of DCs in their spleens. Impaired migration of DCs into the T-cell areas of draining lymph nodes and spleens following CsA-treatment has already been observed^41^. One could speculate that in our model, DCs of BH4-treated mice also displayed impaired migration towards lymphoid organs. Interestingly, an in vitro study showed that an amino-derivate of BH4, rather than BH4 itself, interferes with the antigen-presenting function of DCs. However, we cannot exclude the possibility that the observed effects were related to the redox activity of the compound^42^. Further work are required to clarify this interesting aspect of BH4-treatment in an in vivo model.

In addition to the modulation in the frequency of innate and adaptive immune cells, we observed a modification of cytokine expression in the allograft tissue and in the sera of BH4-treated mice. IL-10 was significantly upregulated in the allograft and sera of BH4-treated mice compared to that in untreated allogeneic controls. Furthermore, IL-10 levels were significantly increased in pan T cells incubated with sepiapterin in vitro. This is in line with the observation that IL-10 inhibits ischemia/reperfusion injury^43^, extends graft survival and function^44–47^ and is essential for the action of Tregs in mediating tolerance in some transplant models^48^.

In our study, IL-4 and IL-5, two main cytokines of the TH2 response, and IL-9, linked to TH2 cells but also produced by Tregs^39,49^, were significantly increased in the allografts (IL-4 and IL-9) and sera (IL-4 and IL-5) of BH4-treated mice. Similarly, GATA-3, the transcription factor that identifies TH2 cells, was also significantly increased in the BH4-treated group compared to that in untreated allogeneic-transplanted animals. In in vitro experiments, pan T cells stimulated with sepiapterin expressed higher levels of IL-4 and IL-13 than those without stimulation. TH2 responses have been shown to promote transplant tolerance^50,51^. Furthermore, TH2 cytokines are known to attenuate the severity of allograft rejection by inhibiting TH1-mediated cytotoxic T lymphocyte and delayed-type hypersensitivity responses. Although tolerizing immunosuppressive therapies often downregulates TH1 but not TH2 responses^52,53^, TH2 cytokines per se are not indicators of graft survival. In this regard, while there are studies that demonstrate a protective effect of TH2 cytokines on the allograft^54–58^ others have proposed the balance between TH1 and TH2 cytokines as a crucial factor affecting the function and type of Tregs^59–62^.

Notably, in the allografts of BH4-treated animals, although significantly decreased compared to allogeneic controls, IL-2 expression was still detected to a sufficient extent, which is of utmost importance for Treg function and proliferation^63–71^.

Moreover, the expression of the pro-inflammatory cytokines TNF-α and to a lesser extent, IL-6 was reduced compared with that in untreated allogeneic controls suggesting a modulation towards a TH2 like cytokine milieu. Interestingly, in the graft, the classical TH1 cytokine IFN-γ was not affected by BH4 or sepiapterin treatment in our in vivo and in vitro models and the transcription factor for the development of TH1 cell expression of T-bet was in the range of untreated allograft controls. So, rather than a switch from TH1 to TH2 BH4 or sepiapterin treatment might induce a modulation of the balance between TH1 and Th2 cytokine. It is worth mentioning that in renal transplant recipients Tregs have been shown to produce IFN-γ^72,73^ and could therefore, at least in part, be responsible for the observed IFN-γ.

In conclusion, our data suggest that the immunosuppressive role of BH4 relies on influencing the interaction between the innate and adaptive immune systems, the proliferative effect on Tregs and mast cells, and the modulation of the cytokine balance. The treatment target of BH4 in acute allograft rejection has not yet been established. However, in light of the already approved treatment with the orally active synthetic BH4-analogon sapropterin dihydrochloride for patients with BH4-deficient phenylketonuria^74^, further dissection of the observed immunomodulatory property could pave the way towards a new clinical application for BH4 designed to exploit potential synergistic effect with CsA.

## Supplementary Information

Below is the link to the electronic supplementary material.

Supplementary Material 1

## Data Availability

The datasets analyzed during the current study are available from the corresponding author on reasonable request.

## Abbreviations

BH2: Dihydrobiopterin
BH4: Tetrahydrobiopterin
b.w.: Body weight
CFSE: Carboxyfluorescein-diacetate-succinimidyl-ester
CsA: Cyclosporin A
CTV: Cell-tracer-violet
DCs: Dendritic cells
GTP: Guanosine triphospahte
i.m.: Intramuscular
i.p.: Intraperitoneal
MACS: Magnetic activated cell sorting
MCs: Mast cells
MLR: Mixed lymphocyte reaction
OD: Once daily
pod: Postoperative day
t.i.d.: Ter in die (three times a day), Tregs, regulatory T cells
Tresp: T responder cells
TPH-1: Tryptophan hydroxylase-1
