# Supplementary material for: Tetrahydrobiopterin enhances regulatory T- and mast cell proliferation and alters cytokines expression in a murine heart transplant model
Source: Sci Rep. 2025 Oct 16;15:36278. doi: 10.1038/s41598-025-20127-1 (PMC12533058; doi:10.1038/s41598-025-20127-1)
Supplement: Supplementary file 1 — Supplementary Material 1 [file 41598_2025_20127_MOESM1_ESM.pdf]

Supplemental Figure 1

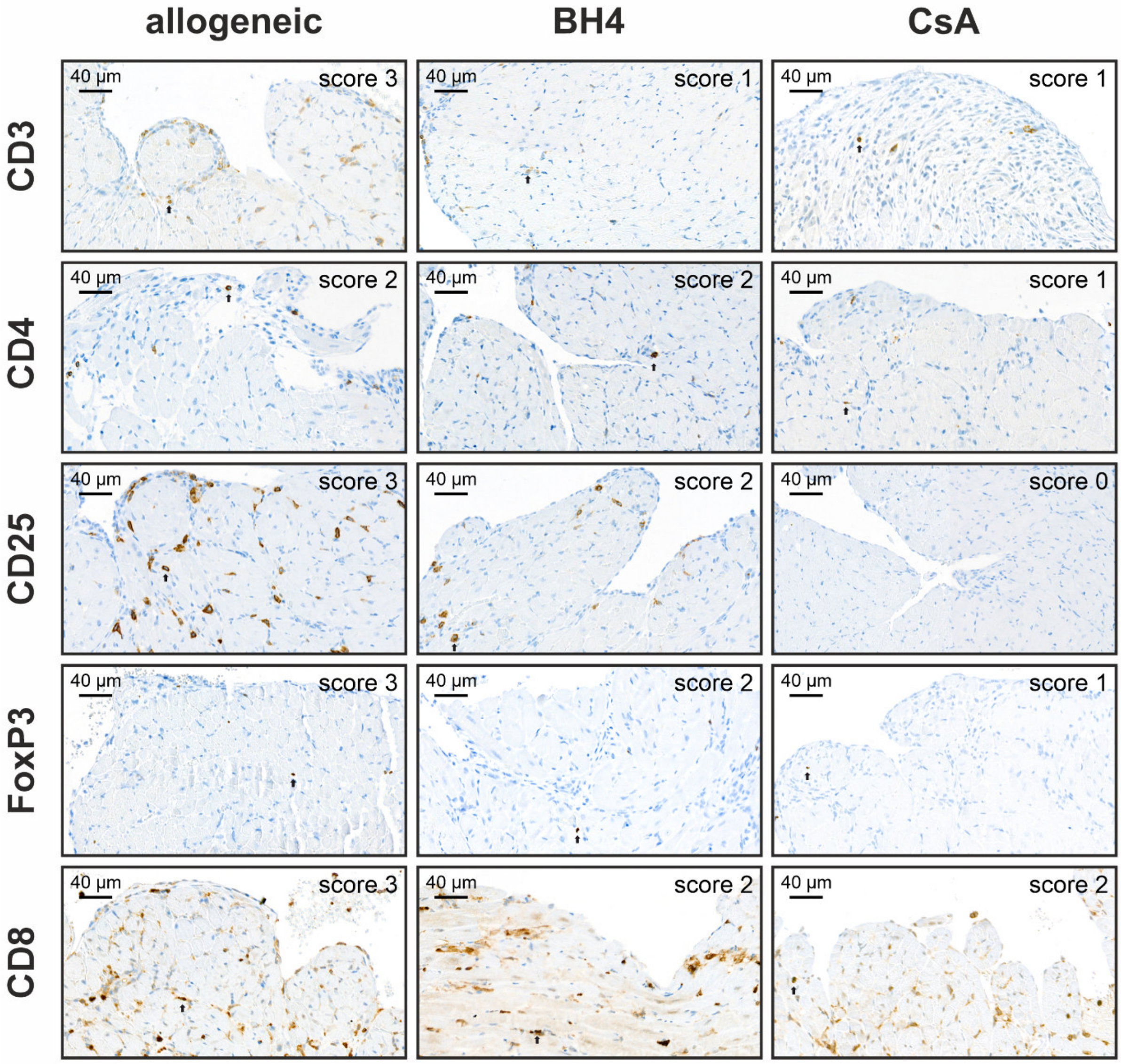

<u>Supplemental Figure 1:</u> Exemplary IHC stainings of graft infiltrating immune cells corresponding to Figure 1 D-H

Supplemental Figure 2

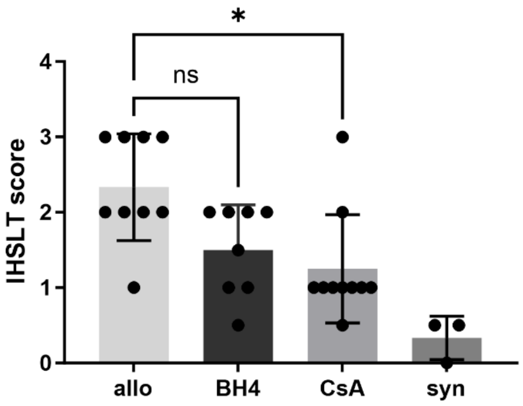

Supplemental Figure 2: ISHLT score of transplanted hearts. Cellular infiltrates and myocyte damage of the grafts were significantly attenuated in CsA-treated compared to untreated animals. BH4 treatment also resulted in lower ISHLT scores, however, without reaching statistical significance. As expected, minimal changes were seen in syngeneic controls. Statistically significant differences between groups were tested applying the Kruskal-Wallis test with a Dunn´s post doc test. Results are presented as Mean + SD. N = 10-3/group. Allogeneic and syngeneic controls showed significant difference (not depicted).

Supplemental Figure 3

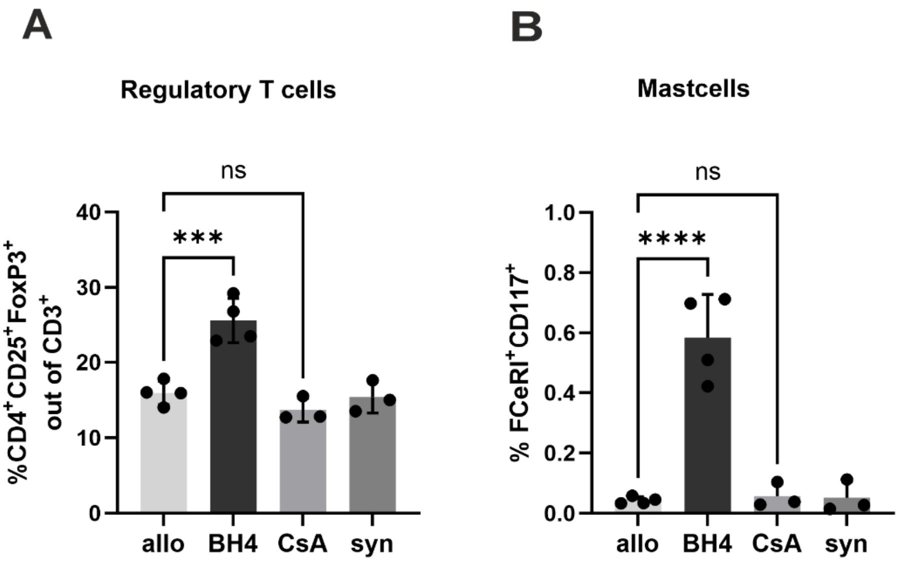

Supplemental Figure 3: Treatment effects on immune cell mobilization in the draining lymph nodes of transplanted grafts. Compared with untreated allogeneic control mice significantly higher frequencies of (A) regulatory T cells and (B) mast cells were detected in lymph nodes of BH4-treated mice. CsA-treated animals showed no increase of regulatory T cells and mast cells compared to untreated allogeneic controls. Statistically significant differences between groups were tested applying the one-way ANOVA test with a Dunnett´s multiple comparison test. N = 3-4/group. Allogeneic and syngeneic controls showed no significant difference (not depicted).

Supplemental Figure 4

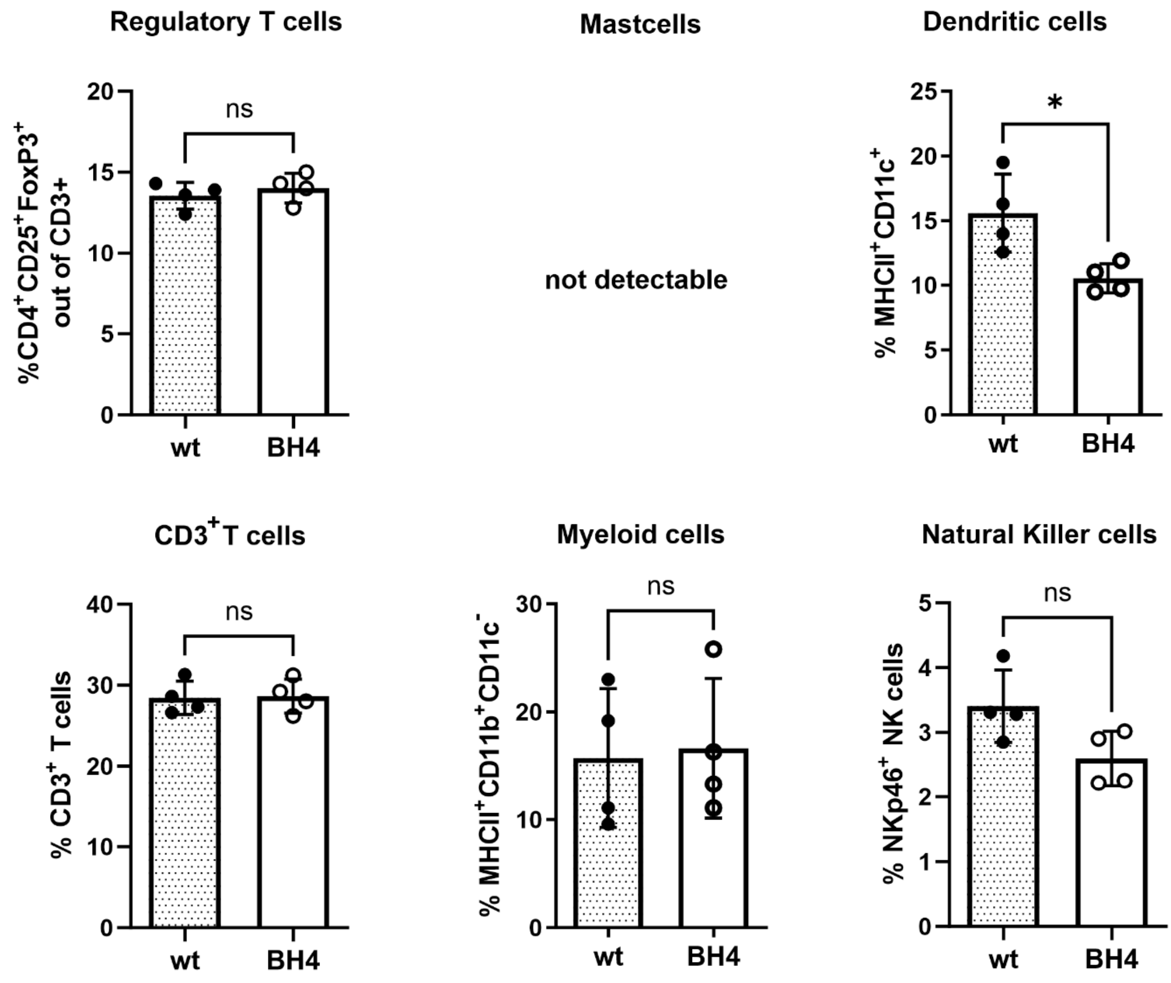

Supplemental Figure 4: Analysis of immune cells in the spleen of untreated wild type (wt) mice and BH4-treated mice without transplantation. The mice were treated identically as recipients of heart transplants treated with BH4. $CD3^{+}$ T cells, NK cells, regulatory T cells and $CD11b^{+}CD11c^{-}$ myeloid cells showed no different frequencies between the two groups. DCs are decreased in BH4-treated animals compared to control mice. Mast cells were not reliably detectable. Differences were analyzed via an unpaired t test, $^{*}p < 0.05$.

Supplemental Figure 5

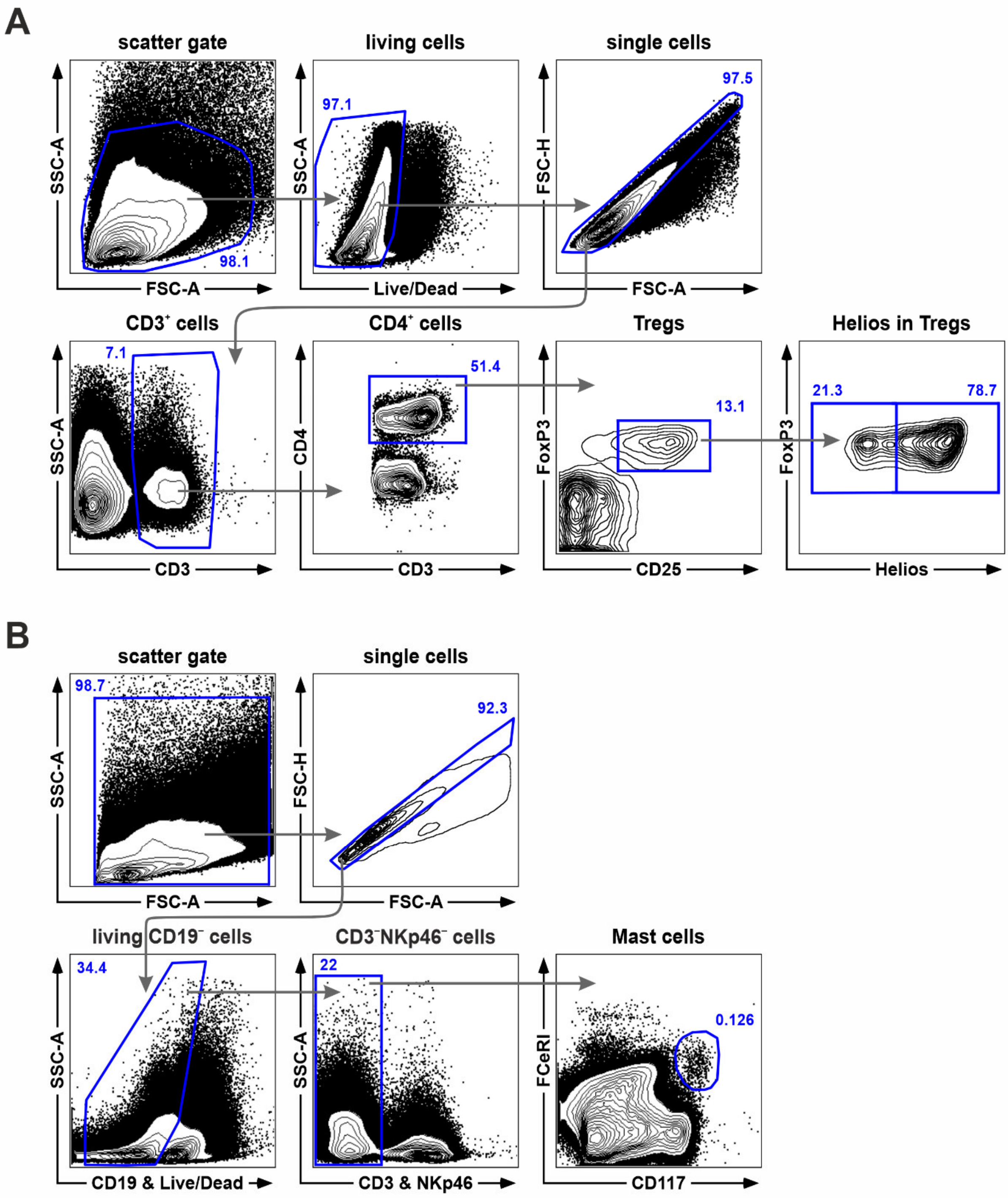

Supplemental Figure 5: Exemplary gating strategy for $FoxP3^+$ regulatory T cells **(A)** and $FCeRI^+CD117^+$ mast cells **(B)** isolated from murine spleens.

Supplemental Figure 6

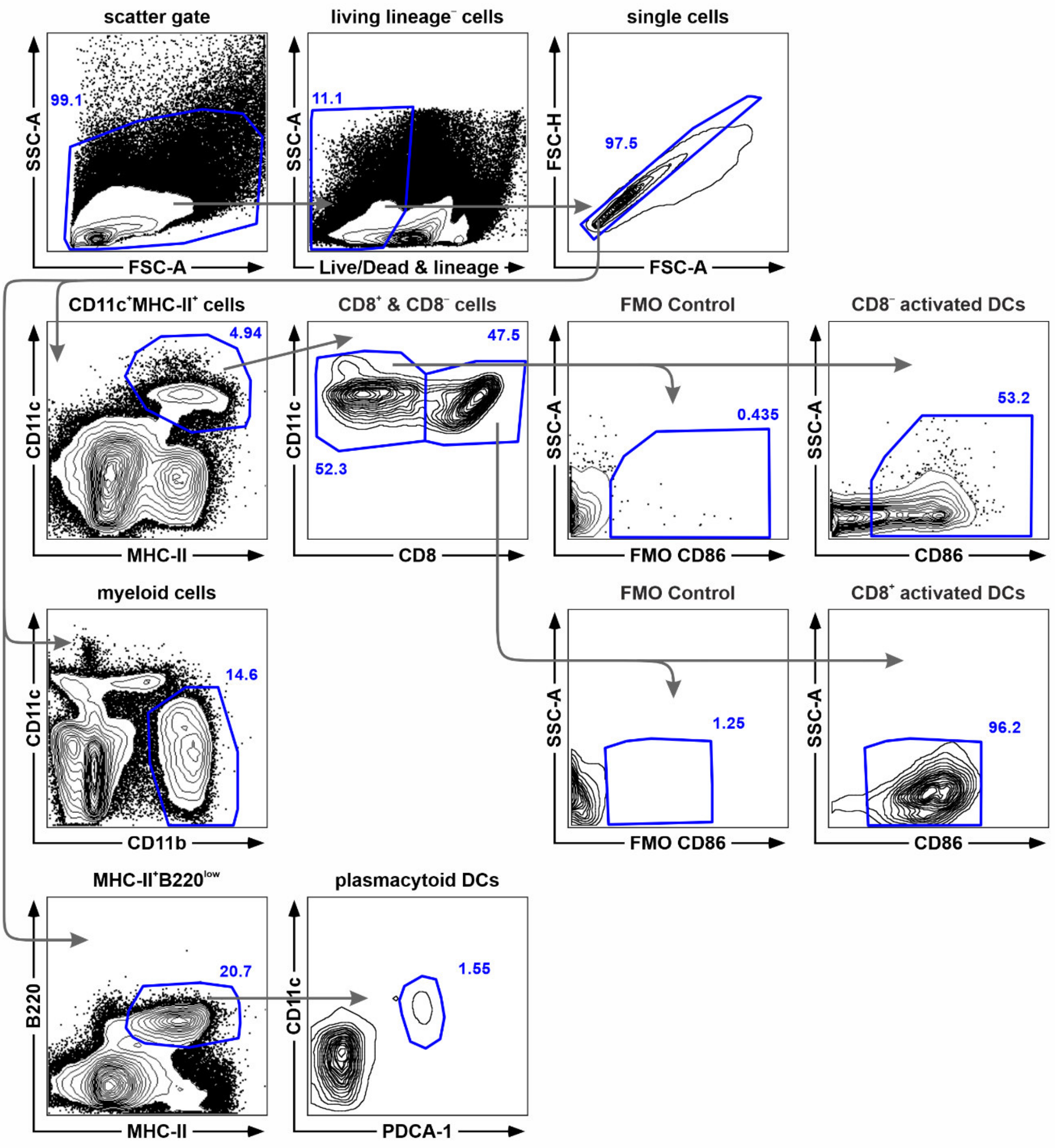

Supplemental Figure 6: Exemplary gating strategy for MHC-II$^+$CD11c$^+$ activated dendritic cells (DCs), PDCA-1$^+$ plasmacytoid dendritic cells and CD11b$^+$CD11c$^-$ myeloid cells isolated from murine spleen. Lineage markers used for exclusion are CD3, CD19 and Nkp46. Dead cells and linage positive cells are depicted in the same fluorescence channel.

Supplemental Figure 7

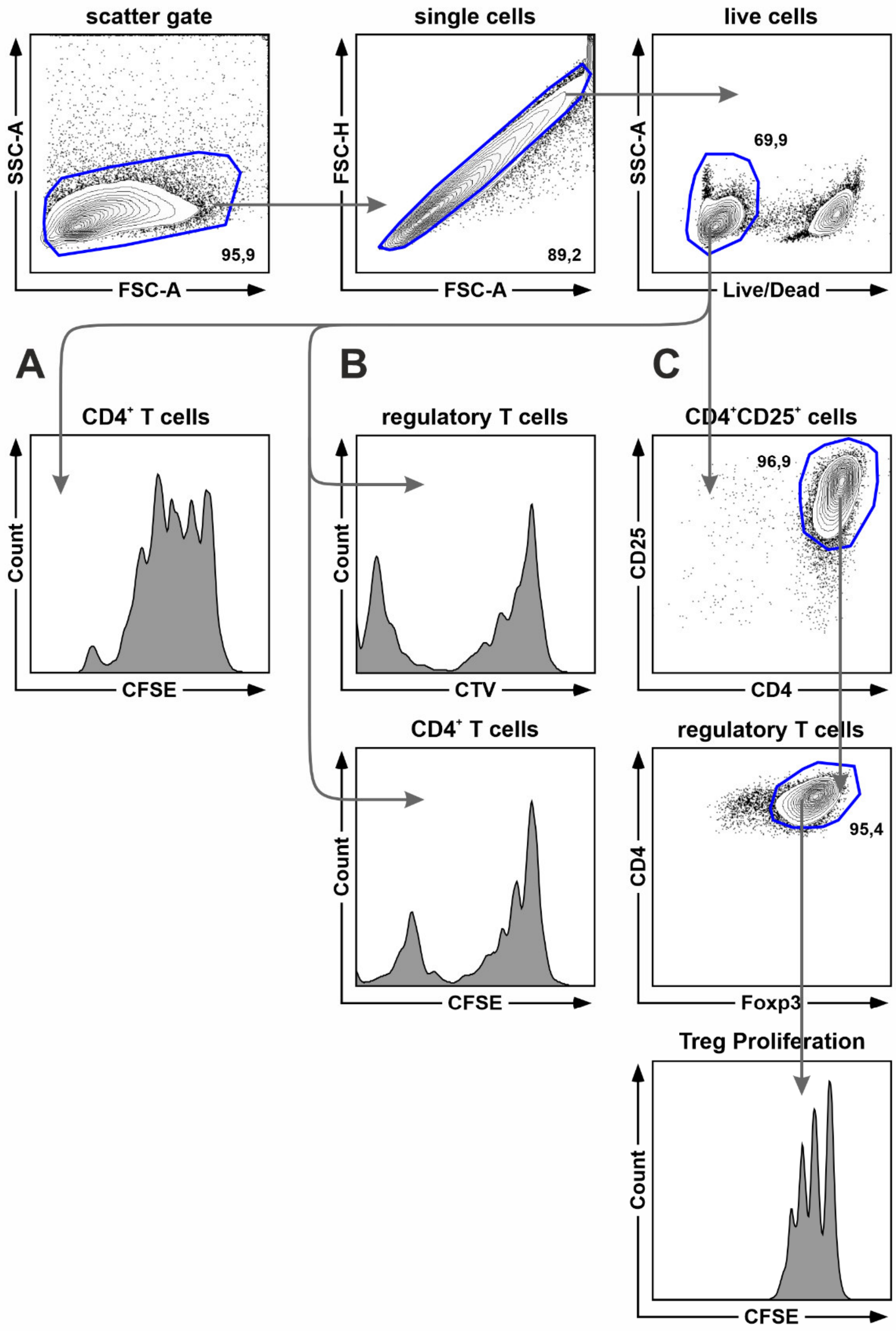

Supplemental Figure 7: (**A**) Gating strategy for the CFSE labeled $CD4^+$ T cells in the mixed lymphocyte reaction (MLR). (**B**) Gating strategy for CFSE labeled $CD4^+$ T cells and CTV labeled regulatory T cells (Tregs) in a T cell suppressor assay. (**C**) Gating strategy for the proliferation assay of CFSE stained Tregs. In the MLR and suppressor assay living cells were selected through a live/dead dye and proliferation of CFSE stained $CD4^+$ T cells and CTV stained Tregs is depicted as histograms. In the proliferation assay $CD4^+CD25^+FoxP3^+$ Tregs were gated after doublet and dead cell exclusion and CFSE staining is shown as a histogram.

Supplemental Figure 8

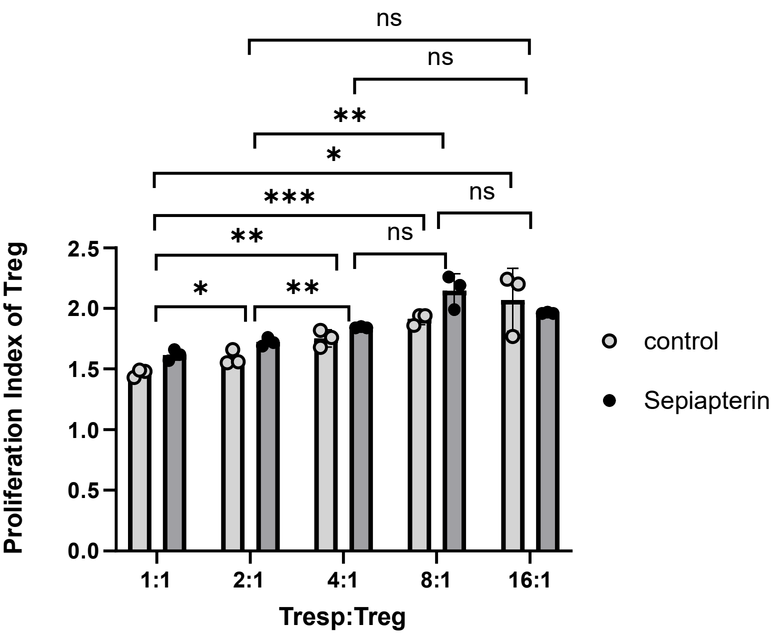

Source of Variation	% of total Variation	P value	P value summary	Significant?	Geisser-Greenhouse´s epsilon
+/- Sepiapterin x Tresp:Treg	6.432	0.0545	ns	No	
+/- Sepiapterin	74.84	0.0003	***	Yes	0.4419
Tresp: Treg	5.010	0.1134	ns	No	
Subject	4.907	0.1118	ns	No	

Supplemental Figure 8: Sepiapterin enhances Treg proliferation independently of Tresp:Treg ratio. Treg proliferation was assessed in suppression assays at different Tresp:Treg ratios (1:1, 2:1, 4:1 , 8:1, 16:1) in the presence or absence of sepiapterin. Bars represent the mean proliferation index of Tregs ± SEM, with individual data points shown for each condition (open circles: control; filled circles: sepiapterin-treated). Statistical comparisons between treatment groups at each ratio were performed using paired t-tests with Holm-Šídák correction for multiple comparisons (*$p < 0.05$, **$p < 0.01$, ***$p < 0.001$, ns = not significant). A two-way repeated measures ANOVA was performed to assess the effects of sepiapterin treatment, Tresp:Treg ratio, and their interaction on Treg proliferation. The summary table shows the percentage of total variation explained, p-values, significance, and Geisser-Greenhouse's epsilon for sphericity correction. Sepiapterin treatment had a significant main effect on Treg proliferation ($p = 0.0003$), while no significant interaction with the Tresp:Treg ratio was observed ($p = 0.0545$), indicating that the enhancement of Treg proliferation by sepiapterin is independent of the suppressive context.

Supplemental Table 1. Antibodies for flow cytometry.

Antibody	Clone	Company
CD3	145-2C11	BDBiosciences
CD4	RM4-5	BDBiosciences
CD8a	53-6.7	BDBiosciences
CD11b	M1/70	BDBiosciences
CD11c	HL3	BDBiosciences
CD19	1D3	BDBiosciences
CD25	PC61	ThermoFisher
CD28	37.51	BDBiosciences
CD44	IM7	BDBiosciences
CD45RA	RA3-6B2	BDBiosciences
CD62L	MEL-14	BDBiosciences
CD64	X54-5/7.1	BDBiosciences
CD86	GL1	BDBiosciences
CD117	2B8	ThermoFisher
CD279	J43	BDBiosciences
CD317	Jf05-1c2.4.1	Miltenyi
FCeR1	MAR-1	ThermoFisher
FoxP3	FJK-16s	ThermoFisher
Helios	22F6	BDBiosciences
MHC class II I-A/I-E	M5/114.15.2	BDBiosciences
NKp46	29A1.4	BDBiosciences
7-AAD		BDBiosciences
FcR-Bloc		BDBiosciences
Fixable Viability Dye eFluor 506		ThermoFisher

Supplemental Table 2. PCR primers

Gene	Assay on demand (Thermo Scientific)
HPRT	Mm03024075_m1
Gata-3	Mm00484683_m1
IL-2	Mm00434256_m1
IL-4	Mm00445259_m1
IL-5	Mm00439646_m1
IL-6	Mm00446190_m1
IL-9	Mm00434305_m1
IL-10	Mm01288386_m1
IL-21	Mm00517640_m1
IL-33	Mm00505403_m1
IFN-γ	Mm01168134_m1
T-bet	Mm00450960_m1
TGF-β	Mm01178820_m1
TIM-3	Mm00454540_m1
TNF-α	Mm00443258_m1
